# Naturally Occurring Microbiota Associated with Mosquito Breeding Habitats and Potential Parasitic Species against Mosquito Larvae: A Study from Gampaha District, Sri Lanka

**DOI:** 10.1155/2020/4602084

**Published:** 2020-04-28

**Authors:** H. A. K. Ranasinghe, L. D. Amarasinghe

**Affiliations:** Department of Zoology and Environmental Management, Faculty of Science, University of Kelaniya, Dalugama, Kelaniya, Sri Lanka GQ 11600

## Abstract

A mosquito species has its own favourable requirements of abiotic and biotic characteristics including microbiota, in a breeding habitat. Some of the microbiota may cause parasitic or pathogenic effects to mosquito larvae such as species of viruses, parasitic bacteria, fungi, protists, entomopathogenic nematodes, and filamentous fungi. In Sri Lanka, there is a scarcity of information on microbiota associated with mosquito breeding habitats and their effect on mosquito larvae. Hence, the present study was conducted to determine microbiota species/taxa associated with a variety of mosquito breeding habitats in selected areas of the Gampaha District in Sri Lanka and the relationship, if any, the microbiota has with mosquito larva survival and breeding. Forty-five microbiota species belonging to 11 phyla were found from different mosquito breeding habitats with the highest percentage belonging to phylum Euglenozoa (27.89%). Species that belonged to the phylum Amoebozoa (1.22%) and Sarcodina (1.17%) had the lowest abundance, and each of its species richness was recorded as one. *Philodina citrina* followed by *Monostyla bulla* comprised 30.8% and 16.59%, respectively, of the total rotifer population. From the total microbiota, 25-50% existed as accidental while less than 25% rare, in the habitat type according to their abundance. Paddy fields had the highest species richness (17), evenness (23.52), Shannon-Weiner (66.64), and beta diversity (0.65) over 50% indicating high heterogeneity in microbiota composition among the habitats. Ciliated protists, namely, *Vorticella microstoma*, *Zoothamnium* spp., and *Chilodinella* sp., were identified as naturally occurring microbiota associated with *Culex* mosquito larvae that inhabited in paddy fields and associated irrigation canals. Only *Vorticella microstoma* caused a significant lethal effect on mosquito larvae. This study revealed that species of *Cx. gelidus*, *Cx. pseudovishnui*, *Cx. tritaeniorhynchus*, *Cx. quinquefasciatus*, and *Cx. whitmorei* served as hosts for *V. microstoma* where infectivity rate in *Cx. tritaeniorhynchus* reached 73.22. *Chilodinella* sp. selectively served as endoparasitic to *Cx. gelidus* larvae causing only 4.58% mortality, and invasive cysts of the pathogen were observed in the subcuticular layer of the host body. Even though *Zoothamnium* spp. were found on *Cx. tritaeniorhynchus* larvae, there was no lethal effect due to the attachment of the parasitic agent. The potential of these microbiotas in integrated vector controlling approaches in future perspectives is recommended.

## 1. Introduction

Distribution, abundance, and individual fitness of mosquito immatures in a particular breeding habitat are known to be dependent on mainly three factors: biotic [1, 2], abiotic [3–5], and their interaction between each other and with other associated taxa [6, 7]. When there is coexistence or mutualism of different mosquito species along with other biotic organisms, they form a community sharing habitat requirements [8]. There are “competitors” of mosquitoes such as algae, bacteria, detritus, and protists that feed upon the same functional food as mosquito larvae in the same habitat [9]. Controphic competitors cause a negative impact on mosquito larval populations. Further, there is an interspecific resource competition under food-limiting environments when multiple mosquito species present simultaneously within the same mosquito breeding habitats [10]. Competitors of mosquito larvae included cladocerans and copepods such as calanoids and harpacticoids [11, 12]. Naturally occurring microcrustaceans could be used as effective competitors against mosquito larvae because many species show similar biotope preferences with mosquito larvae such as early colonization of temporary ponds and filter feeding behavior [13]. Competitors and predators can reduce the survival of mosquitoes either by competing for the same food resources or preying on mosquito larvae. Thus, the interaction of competition and predation of many other invertebrate taxa such as Crustacea, Acaria, and insect larvae who share the same habitats with mosquito larvae is another factor determining the abundance of mosquito larvae in a particular habitat [14, 15]. The major controphic competitors such as cladocerans and ostracods exhibit polyphagous activities with larvae and an effect on their abundance in breeding habitats. Cladocerans are the dominant microinvertebrate which coinhabit with mosquito larvae and other zooplankton communities in rock pools [16]. However, ostracods act as both food competitors and predators of mosquito larvae while copepods act as omnivorous filter feeders which consume mostly large-sized food particles [17]. There are only very few studies and scattered information focused on microbiota association with mosquito larvae in Sri Lanka [18, 19]. Simultaneously, there is a need to develop biopesticides against vector mosquito larvae as a useful substitute to chemical insecticides. In this contest, information on microbiota species association with vector mosquito breeding habitats as potential parasitic or pathogenic species against mosquito immature stages in Sri Lanka should be further studied. Therefore, the present study was conducted to identify naturally occurring microbiota species associated with a variety of vector mosquito breeding habitats and to identify potential parasitic or pathogenic microbiota on mosquito larvae under the natural environment.

## 2. Methodology

### 2.1. Study Area

Gampaha District is located in the west of Sri Lanka and has an area of 1,387 square kilometers. It is bounded by Kurunegala and Puttalam districts from the north, Kegalle District from the east, Colombo District from the south, and the Indian Ocean from the west. The climate is tropical in the Gampaha District with a significant rainfall even in the driest months. The average annual temperature in Gampaha is 27.3°C. In a year, the average rainfall is 2398 mm.

### 2.2. Sampling of Mosquito Breeding Habitats for Microbiota and Mosquito Larvae

**Figure 1:**
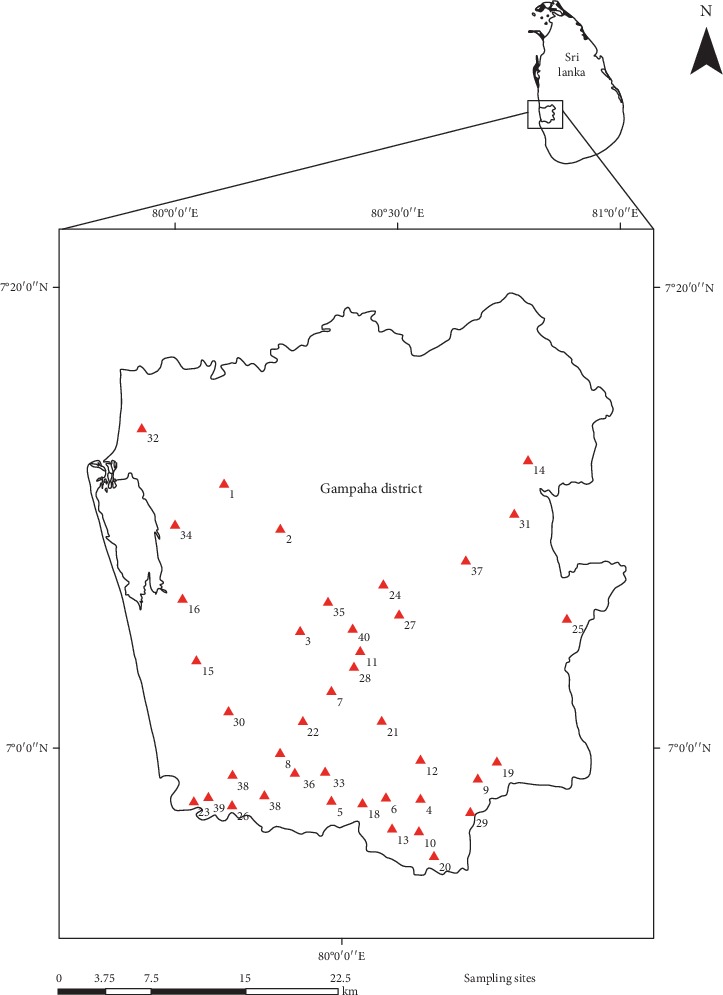
Sampling locations from the selected study site; Gampaha District.

Forty mosquito breeding sites were selected within the district randomly, and each sampling site was georeferenced (GARMIN-etrex SUMMIT) (Figure 1). Water samples from each site were collected using a standard 250 mL dipper bimonthly from September 2017 to August 2018. When dipping is impossible, sampling was performed using pipetting or siphoning methods (maximum 250 mL) into a larval rearing container (height 12 cm × diameter 6.5 cm). Five to eight numbers of mature larvae in a water sample were carefully separated at the site, into a glass vial with 70% ethanol, by using a pipette and labeled for mosquito species identification, and larvae in each sample were identified into species level using standard identification keys [20–22] in the laboratory.

A water sample was then transferred equally into three plastic containers (6.5 cm width, 12 cm height). Two of them were immediately preserved separately in Rose Bengal stain (5% formalin with 0.04% Rose Bengal stain) solution and 5% Lugols' solution for microbiota identification. The remaining sample was kept as nonpreserved and covered with a small-sized mesh net for live observations. All samples were labeled and transferred carefully into the laboratory for further processing.

### 2.3. Identification of Microbiota

One mL aliquot of the preserved sample was examined under a compound microscope (×100 magnification) (OLYMPUS x C21) using a Sedgwick Rafter (S-R) cell (50 mm length, 20 mm width, and 1 mm deep) and HYDRO-BIOS phytoplankton chamber (dimensions, 33 × 33 mm; thickness, 1 mL) for quantifying the microbiota. The sample was well shaken before taking the aliquot for observation. Microbiota species/taxa were recorded, and identification was done to taxa/species level using temporary slide mounts observed under (×400 magnification) using standard identification keys [23–25].

### 2.4. Microbiota Interaction with Mosquito Larvae

Each nonpreserved sample was observed microscopically in a regular manner in the laboratory for microbiota interaction with mosquito larvae until the pupation of mosquito larvae and any observations were recorded.

### 2.5. Data Analysis

Occurrence frequencies of microbiota species were categorized as constant for species found in more than 50% of the collections, common when found between 25% and 50% of the collections, and accidental or rare species when found in less than 25% of the collections [26]. Microbiota alpha diversity (*α*) was calculated for each breeding habitat type as the total number of species in the sampling periods. *α* medium was calculated as the average between the *α* diversity for the system of the same type; gamma diversity (*ϒ*) was estimated using the total number of species from all samples.

Beta diversity (*β*) was estimated by measuring the species turnover using the *β* − 1 index [27], measures the amount that regional diversity exceeds mean alpha diversity, and is calculated by the formula *β* − 1 = [(*S*/*α*mean) − 1]/[*N* − 1] × 100, where *S* is the regional diversity or total richness (the number of species per each sampling site); *α*mean is the mean alpha diversity (mean number of species) for each site in each period; and *N* is the number of sites of the period. Beta diversity over 50% indicates high heterogeneity in microbiota composition among systems; between 20 and 50% indicates intermediate heterogeneity; and below 20% indicates low heterogeneity [27, 28].

The microbiota species diversity was also estimated according to the indices of Shannon and Weaver [29] and evenness [30].

(1) $$\begin{array}{c} \text{Shannon Index }\left(H\right)=-\overset{s}{\underset{i=1}{\sum }}p_{i}\;\ln\;p_{i}, \end{array}$$

In the Shannon index, *p* is the proportion (*n*/*N*) of individuals of one particular species found (*n*) divided by the total number of individuals found (*N*), ln is the natural log, *Σ* is the sum of the calculations, and *s* is the number of species.

Pielou's evenness (*J*)

(2) $$\begin{array}{c} J=\frac{H^{\prime }}{H_{\max}} \end{array}$$

compares the Shannon-Wiener diversity value (*H*′) to the maximum possible diversity value (*H*_max_).

## 3. Results

### 3.1. Diversity and Occurrence of Mosquito and Microbiota Species

**Figure 2:**
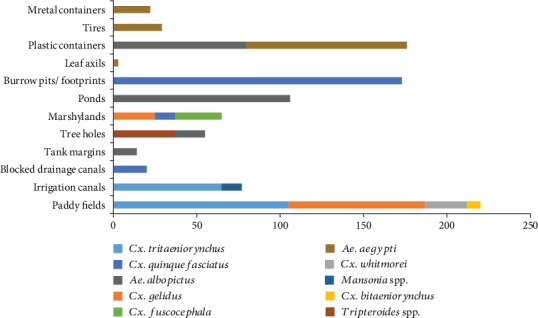
The abundance of mosquito larvae in mosquito breeding habitats from the Gampaha District.

During the study, ten mosquito species from twelve different types of habitats (Figure 2) (paddy fields (*n* = 6), irrigation canals (*n* = 3), blocked drainages (*n* = 1), marshy lands (*n* = 4), tree holes (*n* = 3), tank margins (*n* = 1), plastic containers (*n* = 2), burrow pits/footprints (*n* = 3), ponds (*n* = 1), leaf axils (*n* = 1), used tires (*n* = 1), and metal containers (*n* = 1) were encountered. Six permanent macrotype mosquito breeding habitats, namely, paddy/rice fields, irrigation canals, blocked drainages, marshy lands, ponds, and tank margins, and six temporary microtype mosquito breeding habitats, namely, tree holes, plastic containers, burrow pits/footprints, metal containers, discarded tires, and leaf axils, were found across the study area *Aedes aegypti* and *Aedes albopictus* which were dengue vector mosquitoes in Sri Lanka were prominently found from plastic and metal container habitats while four *Culex* species (*Culex bitaeniorhynchus*, *Culex tritaeniorhynchus*, *Culex gelidus*, *and Culex whitmorei*) were found from rice fields. The highest mosquito abundance was recorded from rice fields.

**Figure 3:**
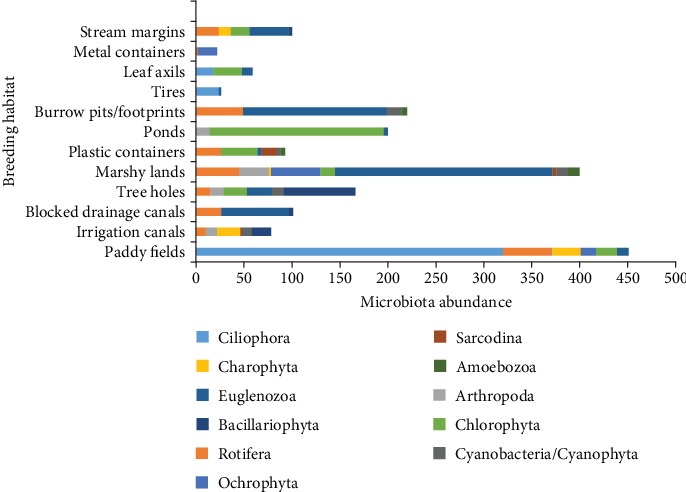
Occurrence of microbiota phyla encountered from different mosquito breeding habitats.

**Figure 4:**
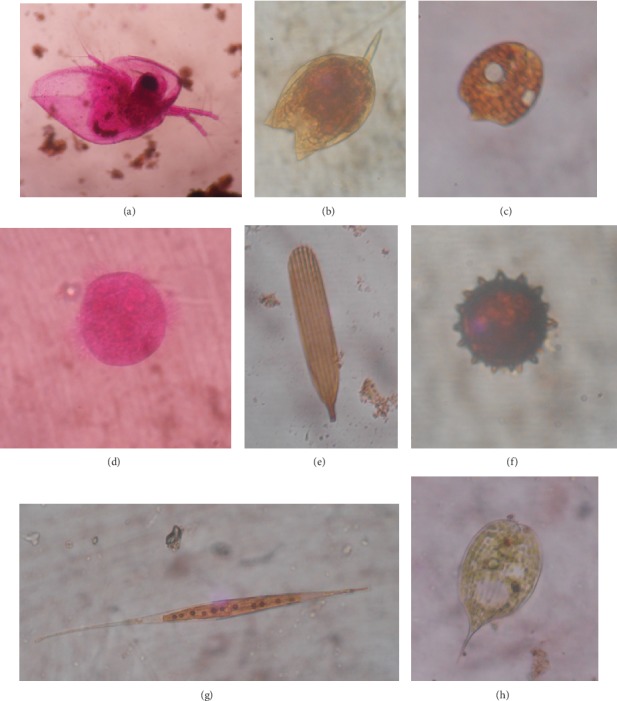
Microscopic view of some microbiota species encountered from mosquito breeding habitats, ×400 magnification (a) *Sida crystallina*, (b) *Lecane luna*, (c) *Phacus pleuronectes*, (d) *Acanthocystis aculeata*, (e) *Notholca acuminata*, (f) *Volvox aureus*, (g) *Closterium* sp., and (h) *Phacus longicauda*.

A total number of 45 microbiota species belong to 11 phyla, namely, Amoebozoa, Arthropoda, Bacillariophyta, Ciliophora, Charophyta, Chlorophyta, Protista, Cyanobacteria/Cyanophyta, Euglenozoa, Ochrophyta/Heterokontophyta, and Rotifera, were recorded from different mosquito breeding habitats (Figure 3). The highest percentage abundance was recorded from members of the phylum Euglenozoa (27.89% of total microbiota) while the highest number of species was recorded from members of phylum Rotifera (Table 1). Among them, *Philodina citrina* followed by *Monostyla bulla* comprised 30.8% and 16.59%, respectively, of the total rotifer population. They exhibited a very wide range of morphological variations (Figure 4). Species of the phylum Amoebozoa and Sarcodina had the lowest abundance, and each of its species richness was recorded as one. *Phacus pleuronectes* (81.97%) in burrow pits/footprints, *Gloeocystis gigas* (90.91%) in a metal container, *Paramecium bursaria* (92.31%) in discarded tires, *Volvox aureus* in leaf axils (50.85%), and *Pediastrum biradiatum* (53.33%) in ponds existed as constant species in the particular breeding habitat (Table 2). *Vorticella microstoma* (42.28%) and *Zoothamnium* spp. (25.37%) existed as common species in paddy fields. *Cosmarium quadricauda* (30.77%) in irrigation canals, *Phacus pleuronectes* (47.71%) in blocked drainages, *Phacus caudatus* (31.38%) in marshy lands, and *Paramecium bursaria* (30.51%) in leaf axils also existed as common species. Additionally, plastic containers (*Philodina citrina* 26.88%, *Scenedesmus bijuga* 32.26%) and stream margins (*Phacus pleuronectes* 26.7%, *Arthrodesmum incus* 33.33%) had two common microbiota species in each of their habitats. However, the majority of the microbiota existed as accidental or rare species in the habitat type according to their abundance (Table 2).

Species richness of the microbiota was highest in paddy fields (Table 2; gamma diversity, *ϒ*). Paddy fields had the highest beta diversity over 50% indicating high heterogeneity in microbiota composition among the systems. Irrigation canals, tree holes, marshy lands, plastic containers, and burrow pits/footprints had beta diversity between 20% and 50%, indicating intermediate heterogeneity in microbiota composition among the systems. Blocked drainages, ponds, metal containers, leaf axils, tires, and tank margins had a beta diversity below 20%, indicating low heterogeneity. Paddy fields resulted the highest Shannon-Weiner diversity index and evenness values.

### 3.2. Parasitic or Pathogenic Microbiota

During the time natural population of mosquito larvae is kept under regular check in the laboratory, unusual high mortalities were observed in *Cx. tritaeniorhynchus* mosquito larvae collected from a paddy field which prompted to detect the causative organism. A peritrich ciliate, *Vorticella microstoma* (Identification key [24]), was found attached to the body of such dead larvae.

**Figure 5:**
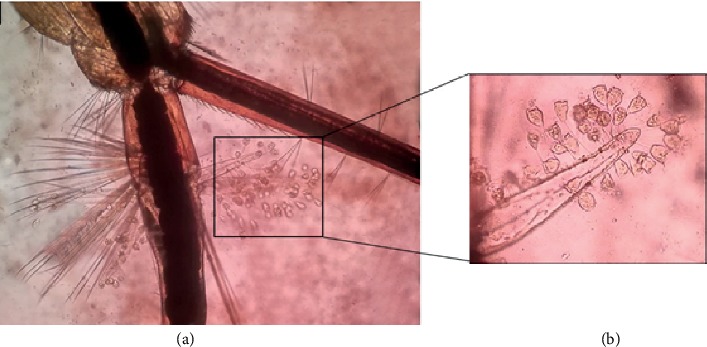
(a) Infestation of *V. microstoma* to 3^rd^ instar larva of *Cx. pseudovishnui* anal papillae region (x40 magnification); (b) attached trophonts of *V. microstoma* (x100 magnification).

Five species of *Culex* mosquito larvae (*n* = 1587) collected from paddy fields (*n* = 24) and associated irrigation canals (*n* = 10) were resulted with varying degrees of *V. microstoma* infestation under natural environmental conditions and are shown in Table 3. Out of the total collection of *Culex* mosquito larvae, 47.07% (*n* = 747) were positive for *V. microstoma* infestation (Figures 5(a) and 5(b)). The infectivity rate (percentage of larvae infested with *V. microstoma*) of *Cx. tritaeniorhynchus* was higher compared with that of the other *Culex* species, which comprised 73.22% of the total collection (Table 3). During the study, *Cx. quinquefasciatus* larvae were found associated in abandoned paddy fields where the parasitic species was not usually detected. However, only 40 out of 108 (37.04%) were found to harbor *V. microstoma*, indicating relatively a low larval susceptibility to ciliate infection compared to other vulnerable *Culex* species (Table 3).

**Figure 6:**
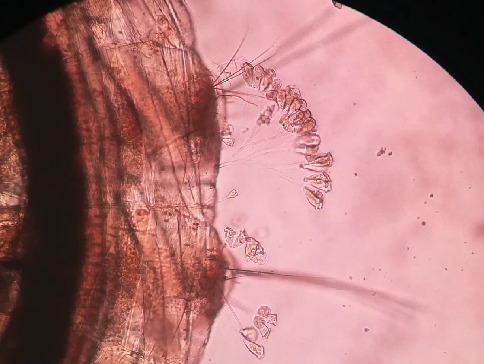
Infection of parasite (*Zoothamnium* sp.) to 3^rd^ larval instars of *Cx. tritaeniorhynchus* abdominal region (x100 magnification).

*Zoothamnium* sp. was recorded as parasitic on *Cx. gelidus* mosquito larvae (Figure 6) in this study but observations did not support for its lethal effect on mosquito larvae. *Zoothamnium* sp. has one main stalk with many branches ending in zooids, which is the distinct morphological feature to distinguish it from *Vorticella*. *Vorticella* has only a single stalk with one zooid. Upon stimulation, *Zoothamnium* entire colony contracts into one large globule and then folds the main stalk.

**Figure 7:**
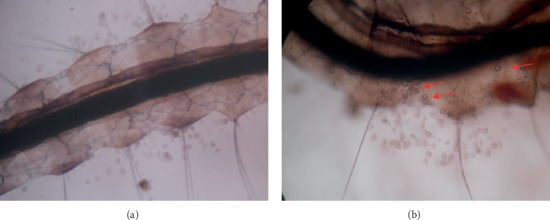
Transparent *Cx. tritaeniorhynchus* larva (magnification x40) showing endoparasitic ciliate, *Chilodinella*, in the host body (a, b); (b) cuticular invasive cysts (arrow marks) of the pathogen on the cuticle of host body (magnification x100).

*Chilodinella* sp. was identified as endoparasitic ciliate causing a pathogenic effect (Figure 7) under natural environmental conditions on *Cx. tritaeniorhynchus* mosquito larvae collected from a paddy field (6°57.959′ N, 79°59.492′ E). However, considerable mortality was not observed (4.58% mortality of larvae compared to controls) due to the infestation of this pathogen to mosquito larvae. Identification was performed by observing the ciliates in wet mounts (Ehrenberg, 1838) (subphylum: Ciliophora: Crytophorida: Chilodonellidae). Endoparasitic ciliates were reported in the host larval body under microscopic observations only.

## 4. Discussion

Endoparasitic ciliate (Protista: Ciliophora), *Lambornella stegomyiae*, was first reported to infect *Aedes albopictus* larvae in a sample collected from an earthen pot in Kuala Lumpur [31]. Micks [32, 33] reported the lethal effect of the ciliate, *V. microstoma*, on *Anopheles quadrimaculatus* and *An. atroparvus*, respectively. Chandrasekar et al. [34] reported that infestation of *Vorticella* sp. on *Anopheles stephensi* larvae has caused an inhibition of larval growth, development, and adult emergence. During the present study, *Vorticella microstoma*, *Zoothamnium* spp., and *Chilodinella* sp. were identified as ciliated parasitic or pathogenic species in this study, causing a lethal effect by *V. microstoma* on *Culex tritaeniorhynchus*, *Cx. gelidus*, *Cx. pseudovishnui*, and *Cx. quinquefasciatus* mosquito larvae. It is important to state that all these ciliates were recorded from paddy/rice field habitats. *V. microstoma* is effective on mainly the paddy field-inhabiting *Culex* mosquitoes. Numerous shallow pools and irrigation canals built by paddy farmers during seedling transplanting usually serve as ideal breeding sites for mosquito larvae and associated ciliates. Mutero et al. [35] stated that application of nitrogen fertilizer to growing paddy further increases its larval densities. Rainfall alters the physicochemical properties of rice fields, resulting in changes in larval densities and species succession. Once the paddy is harvested in dry condition, abandoned vector-paddy field breeding habitats restrict distribution of parasitic or pathogenic ciliates in the absence of host mosquito larvae. Thus, parasitic agents necessarily undergo a quiescent period to overcome dry spell until the next planting season of paddy coupled with monsoon rains with high vector density situation returns. Thus, the encystation of these ciliates seems a possible way for the time lapse. After excystation, the free-swimming trophont stage of these ciliates could be increased rapidly when the optimum environmental conditions are resumed. Cysts and the process of encystation and excystation have been described in detail for the species, *V. microstoma* [36] and *Chilodinella uncinata* ([37]). Even though *Chilodinella* sp. did not cause considerable mortality in *Cx. gelidus* mosquito larvae in the present study, Das [37] reported that species *C. uncinata* has caused 25–100% mortalities in Japanese encephalitis (JE) vector larvae in North India.

The microinvertebrates, namely, *Paramecium caudatum*, *Brachionus forticula*, *Philodina citrina*, *Diaphanosoma brachyurum*, and *Sida crystallina*, were recorded in association with mosquito larvae in dried ponds, marshy lands, and irrigation canals in this study. As reported by Obi et al. (2017), relative abundance of mosquitoes has significantly correlated negatively with those microinvertebrates in rock pools [38]. Garcia-Sánchez et al. [39] reported the presence of three main phyla of algae, Bacillariophyceae, Chlorophyceae, and Cyanobacteria in *Aedes aegypti* larval habitats in artificial water containers in Girardot, Colombia. Similarly, these algal phyla were reported from both natural and man-made breeding habitats in our study. As reported by Addicott [40] and Blaustein and Chase [1], heterotrophic microeukaryotes such as protists and rotifers in breeding habitats particularly in container habitats are important components of nutritional resources for larvae. Twelve species of rotifers, namely, *Lecane lunaris*, *Keratella tropica*, *Lecane luna*, *Lecane papuana*, *Lepadella ovalis*, *Monostyla bulla*, *Notholca acuminata*, *Pandorina morum*, *Philodina citrina*, *Diurella stylata*, *Euchlanis dilatata*, and *Brachionus forficula* were associated with breeding habitats positive for mosquito larvae. Only one species of cyclopoid copepod, *Metacyclops minutus*, was recorded with lower occurrence frequency (0.21%) from paddy fields in this study. However, *M. minutus* in this study did not cause any effect in reduction of larval abundance. Several authors reported that cyclopoid copepods act as effective biocontrol agents of mosquito larvae but under the field conditions, the use of crustaceans has become limited during the initial phase of their community development in which their abundance is low [41–43].

Cyanobacteria play an important role as diet items of mosquito larvae. *Spirulina major* from tree holes, marshy lands, plastic containers, and burrow pits/footprints and *Anabaena affinis* from irrigation canals were recorded as cyanobacteria species. However, species, namely, *Kirchneriella*, *Scenedesmus*, *Coelastrum*, *Selenastrum*, *Dactylococcus*, and *Tetrallantos*, are virtually indigestible by *Culex*, *Aedes*, and *Anopheles* mosquito larvae, hence causing a reduction of larval survival [41]. Marten [41] reported that mosquito larvae failed to develop successfully in the water where certain species of closely related green algae in the order Chlorococcales are the main source of larval food. Howland [44] has reported that *Scenedesmus quadricauda* shows no signs of digestion in the mosquito gut. *S. quadrimaculatus* was recorded from ponds (9.52%) in the present study with no lethal effect on *Ae. albopictus* mosquito larvae found from the same habitat. Two more species of *Scenedesmus* were recorded from this study, namely, *S. armatus* from blocked drainages and tree holes and *S. bijuga* from plastic containers, ponds, and paddy fields with no significant effect on mosquito larvae.

## 5. Conclusions

A total number of 45 microbiota species belong to 11 phyla were encountered from different mosquito breeding habitats during the study while the highest percentage abundance was found from phylum Euglenozoa (27.89%), and species under phylum Amoebozoa (1.22%) and Sarcodina (1.17%) had the lowest abundance, and each of its species richness was recorded as one. The majority of the microbiota existed as accidental (abundance 25-50% of the collections) or rare species (less than 25% of the collections) in the habitat type according to their abundance. Paddy fields had the highest species richness (17), evenness (23.52), Shannon-Weiner (66.64), and beta diversity (0.65) over 50% indicating high heterogeneity in microbiota composition among the systems. The autotrophic protists in genera *Euglena*, *Closterium*, and *Pinnularia* served as the diet items to mosquito larvae. *Vorticella microstoma*, *Zoothamnium* spp., and *Chilodinella* sp. were found as possible parasitic and pathogenic agents against mosquito larvae. *Vorticella microstoma* caused a lethal effect on *Cx. tritaeniorhynchus* larvae while *Cx. tritaeniorhynchus*, *Cx. gelidus*, *Cx.* pseudovishnui, *Cx. quinquefasciatus*, and *Cx. whitmorei* mosquito larvae were found to be infected with *V. microstoma* in natural environmental conditions. However, 4.58% mortality of *Cx. gelidus* larvae were observed while no lethal effect of *Zoothamnium* spp. was found on *Cx. tritaeniorhynchus*.

## Figures and Tables

**Table 1:** Occurrence frequencies of microbiota species in different types of breeding habitats.

Species	Percentage occurrence of microbiota in habitat type											
	Paddy fields	Irrigation canals	Blocked drainage canals	Tree holes	Marshy lands	Plastic containers	Dried up ponds	Burrow pits/footprints	Discarded tires	Metal containers	Leaf axils	Tank margins
Phylum Ciliophora												
*Vorticella microstoma*	42.28^B^	0	0	0	0	0	0	0	0	0	0	0
*Zoothamnium spp.*	25.37^B^	0	0	0	0	0	0	0	0	0	0	0
*Paramecium bursaria*	0	0	0	0	0	0	0	0	92.31^A^	0	30.51^B^	0
Phylum Rotifera												
*Keratella tropica*	0	0	0	0	5.59^C^	0	0	0	0	0	0	0
*Lecane lunaris*	2.11^C^	0	0	0	0	0	4.76^C^	0	0	0	0	0
*Lecane luna*	4.23^C^	0	0	0	0	0	0	0	0	0	0	0
*Lecane papuana*	0	0	3.67^C^	0	0	0	0	0	0	0	0	0
*Lepadella ovalis*	0.85^C^	0	0	0	0	0	0	0	0	0	0	0
*Monostyla bulla*	2.75^C^	0	0	0	0	0	0	5.46^C^	0	0	0	8^C^
*Notholca acuminata*	0	12.82^C^	0	0	0	1.08^C^	0	0	0	0	0	0
*Pandorina morum*	0	0	0	4.13^C^	0	0	0	0	0	0	0	0
*Philodina citrina*	0.42^C^	0	18.35^C^	8.26^C^	0	26.88^B^	0	0	0	9.09^C^	0	3.33^C^
*Diurella stylata*	0.42^C^	0	1.83^C^	0	1.4^C^	0	0	0	0	0	0	2^C^
*Brachionus forficula*	0.85^C^	0	0	0	0	0	0	1.09^C^	0	0	0	0
*Euchlanis dilatata*	1.27^C^	0	0	0	0	0	0	0	0	0	0	2.67^C^
Phylum Cyanobacteria/Cyanophyta												
*Spiriluna major*	0	0	0	9.92^C^	3.35^C^	4.3^C^	0	8.2^C^	0	0	0	0
*Anabaena affinis*	0	12.82^C^	0	0	0	0	0	0	0	0	0	0
Phylum Amoebozoa												
*Arcella arenaria*	0	0	0	0	3.35^C^	5.38^C^	0	3.28^C^	0	0	0	0
Phylum Sarcodina												
*Acanthocystis aculeata*	0	2.56^C^	0	0	1.12^C^	17.2^C^	0	0	0	0	0	0
Phylum Euglenozoa												
*Euglena geniculata*	2.54^C^	0	7.34^C^	20.66^C^	0	0	0	0	0	0	0	0
*Euglena acus*	0	0	9.17^C^	0	0	4.3^C^	1.9^C^	0	7.69^C^	0	0	0
*Phacus pleuronectes*	0	0	47.71^B^	0	19.55^C^	0	0	81.97^A^	0	0	0	26.67^B^
*Phacus caudatus*	0	0	0	0	31.28^B^	0	0	0	0	0	0	0
*Phacus longicauda*	0	0	0	0	6.98^C^	0	0	0	0	0	0	0
*Euglenopsis vorax*	0	0	0	0	0	0	0	0	0	0	18.64^C^	0
Phylum Charophyta												
*Cosmarium obsoletum*	5.92^C^	0	0	0	0	0	0	0	0	0	0	0
*Cosmarium antilopeum*	0.21^C^	0	0	0	0	0	0	0	0	0	0	0
*Cosmarium quadrum*	0	30.77^B^	0	0	0	0	0	0	0	0	0	0
*Closterium spp.*	0	0	0	0	0	0	0	0	0	0	0	8^C^
Phylum Ochrophyta												
*Pinnularia braunii*	0	0	0.92^C^	0	14.53^C^	0	0	0	0	0	0	0
*Gloeobotrys limneticus*	3.38^C^	0	0	0	0	0	0	0	0	0	0	0
Phylum Chlorophyta												
*Crucigenia rectangularis*	0	0	0	0	0	0	0	0	0	0	0	13.33^C^
*Gloeocystis gigas*	0	0	0	0	0	0	0	0	0	90.91^A^	0	0
*Pediastrum biradiatum*	0	0	0	0	0	0	53.33^A^	0	0	0	0	0
*Scenedesmus quadricauda*	0	0	0	0	0	0	9.52^C^	0	0	0	0	0
*Scenedesmus armetus*	0	0	7.34^C^	9.92^C^	0	0	0	0	0	0	0	0
*Volvox aureus*	0	0	0	9.92^C^	4.19^C^	8.6^C^	0	0	0	0	50.85^A^	0
*Scenedesmus bijuga*	4.65^C^	0	0	0	0	32.26^B^	23.81^C^	0	0	0	0	0
Phylum Bacillariophyta												
*Siurella robusta*	0	6.41^C^	3.67^C^	0	0	0	0	0	0	0	0	0
*Gomphonema angustatum*	0	19.23^C^	0	20.66^C^	0	0	0	0	0	0	0	2.67^C^
*Arthrodesmum incus*	0	0	0	0	0	0	0	0	0	0	0	33.33^B^
Phylum Arthropoda												
*Metacyclops minutus*	0.21^C^	0	0	0	0	0	0	0	0	0	0	0
*Canthocamptus staphylinus*	0	15.58^C^	0	16.53^C^	2.23^C^	0	0.95^C^	0	0	0	0	0
*Sida crystallina*	0	0	0	0	6.42^C^	0	0	0	0	0	0	0
*Diaphanosoma brachyurum*	2.54^C^	0	0	0	0	0	5.71^C^	0	0	0	0	0

^A^Constant species; ^B^common species; and ^C^accidental or rare species of the collected samples included in parentheses.

**Table 2:** Evenness, Shannon diversity, alpha (*α*), alpha medium, and beta (*β*) and gamma (*ϒ*) diversities of type of habitats.

Habitat	Alpha diversity	Alpha medium diversity	Beta diversity	Gamma diversity	Shannon-Weiner diversity index	Evenness
Paddy fields	9	4	0.65	17	66.64	23.52
	5					
	3					
	4					
	1					
	4					

Irrigation canals	4	4	0.38	7	15.22	7.82
	4					
	3					

Blocked drainages	9	9	0	9	25.35	11.54

Tree holes	5	5	0.3	8	17.57	8.45
	8					
	3					

Marshy lands	7	5	0.47	12	35.55	14.31
	8					
	5					
	1					

Plastic containers	3	2	0.75	8	20.41	9.82
	2					
	4					
	1					
	1					

Ponds	8	8	0	8	18.94	9.11

Burrow pits/footprints	3	2	0.75	5	13.54	8.41
	1					
	3					

Metal containers	2	2	0	2	2.49	3.59
	2					

Leaf axils	3	3	0	3	3.54	3.22
Tires	2	2	0	2	2.64	3.81

Tank margins	9	9	0	9	24.05	10.95


**Table 3:** Number of mosquito larvae examined and recorded as naturally infested with *V. microstoma* in paddy fields and associated irrigation canals.

Habitat type searched	(Number of mosquito larvae found infected/numbers examined for infection)∗100					
	*Cx. bitaenyorhynchus*	*Cx. tritaeniorhynchus*	*Cx. gelidus*	*Cx. quinquefasciatus*	*Cx. whitmorei*	*Cx. pseudovishnui*
Paddy fields	0/451 = 0	186/254 = 73.22%	143/289 = 49.48%	40/108 = 37.04%	0	120/485 = 24.74%
Irrigation canals	0	0	208/350 = 59.42%	0	50/110 = 45.45%	0

## Data Availability

The datasets supporting the conclusions of this article are included in the article.
